# Machine learning-derived cellular senescence index for predicting prognosis and drug sensitivity in patients with renal cell carcinoma

**DOI:** 10.3389/fimmu.2025.1727099

**Published:** 2025-12-16

**Authors:** Le Meng, Haoxun Zhang, Yifan Qiu, Xiangyu Zhu, Xuran Ji, Bowen Wang, Guoling Zhang, Yue Xue, Chunyang Wang

**Affiliations:** 1 Department of Urology, the First Affiliated Hospital of Harbin Medical University, Heilongjiang, Harbin, China; 2 Urology ward, Jiangsu Province Geriatric Hospital, Nanjing, Jiangsu, China

**Keywords:** ccRCC, immunotherapy, machine-learning, precise medicine, senescence

## Abstract

Cellular senescence, an inevitable phase in the cellular lifecycle, is increasingly implicated in cancer development. Clear cell renal cell carcinoma (ccRCC), a lethal malignancy of the urinary system, underscores the need for senescence-based risk models. Through single-cell analysis, we identified senescent cells within ccRCC tumors and delineated their distinct biological features. We then integrated ten machine learning algorithms—plsRcox, Ridge, Enet, CoxBoost, Lasso, StepCox, RSF, SuperPC, GBM, and survivalSVM—generating 101 combinatorial models via pairwise integration. The optimal Lasso-StepCox model was selected based on the highest mean concordance index (C-index), yielding a minimized senescence-related gene signature of only 9 genes (significantly below the typical 15–30-gene range). This signature formed the basis of a senescence-related scoring model (SRSM) for ccRCC patient survival assessment. Patients with high SRSM exhibited significantly poorer survival (P < 0.001), enhanced oxidative phosphorylation, and an immunosuppressive tumor microenvironment (TME) characterized by elevated regulatory T cell (Treg) infiltration. *In vitro* validation confirmed that NME2 knockdown suppressed ccRCC proliferation and invasion. Collectively, the SRSM framework provides a precise tool for prognostic stratification and therapeutic targeting in ccRCC.

Therefore, to bridge the gap between the intricate landscape of cellular senescence revealed by scRNA-seq and the pressing clinical need for robust prognostic tools, we aimed to develop a quantifiable senescence-related scoring model (SRSM). By integrating multiple machine learning algorithms, we sought not only to achieve superior predictive accuracy but also to derive a minimized gene signature for enhanced clinical applicability, ultimately translating the biological insights of senescence into a precise prognostic framework for ccRCC.

## 1 Introduction

Renal cell carcinoma (RCC) is a prevalent malignancy of the urinary system (1). The World Health Organization states that RCC causes 150,000 to 170,000 deaths annually, and this number is on the rise with social development (2). Clear cell renal cell carcinoma (ccRCC) is the most predominant subtype of RCC, taking up around 70% of RCC cases (3) and the highest mortality rate among these subtypes (4). Currently, surgery is the most common choice for ccRCC, including partial nephrectomy and radical nephrectomy (5). However, due to the difficulty in early diagnosis of advanced ccRCC, even after radical surgery, patients with advanced ccRCC have an overall survival rate of less than 30% (6). With advances in precision medicine, immunotherapy and targeted therapy are gradually gaining popularity (7). So far, many monoclonal antibody drugs and targeted chemotherapy drugs have been developed (8). However, as antitumor drugs are employed, patients are demonstrating significant resistance, which underscores the pressing need to identify precise therapeutic targets for the treatment of clear cell renal cell carcinoma. Cellular senescence occurs at any stage of an organism’s life, specifically the cessation of the cell cycle, and involves many physiological and pathological mechanisms (9). Cellular senescence is an irreversible process in cell proliferation (10), which can induce changes in cell metabolism and usually lead to cell death (11). Therefore, cellular senescence is significant for the organism itself in terms of eliminating harmful cells. Cellular senescence is generally considered an important suppressor of cancer cells (12) as it can limit the tumor itself and activate immune cells through antigens released by senescent cells (13). However, recent reports have indicated senescent cells can evade immune surveillance and clearance by secreting senescence-associated secretory phenotype (SASP) factors, like IL-10 and IL-6 (14). In addition, the senescence and exhaustion of anti-tumor immune cells, such as T cells and NK cells, foster the immunosuppressive tumor microenvironment (TME) (15). Cellular senescence is recognized as a key feature of malignant tumors, where although cell growth stops permanently, the metabolism remains active. During this process, various pro-inflammatory and proteolytic substances are released, which are integral components of SASP (16, 17). In summary, there remains an intricate relationship among cellular senescence, tumor treatment resistance, and worse outcomes. Research has demonstrated that extensive changes in cellular senescence-related metabolic processes are greatly correlated with ccRCC prognosis (18). Given the significant impact of cellular senescence on tumor development and progression, identifying key senescence-related genes (SRGs) is of paramount significance for developing novel targeted therapies for ccRCC.

RNA-single-cell sequencing (scRNA-seq) technology can identify cell types and understand their biological characteristics. Machine learning makes medical research more precise, enabling the identification of genes that play key roles in disease progression from vast amounts of transcriptomic data (19).

The study employed scRNA-seq data analysis to uncover the unique biological characteristics of senescent cancer cells. Then 101 machine learning combinations were integrated to identify the best algorithm for constructing a novel risk model. Finally, target genes were validated through *in vitro* experiments. In the TME, cancer cells exhibit significantly high levels of senescence, and those cells that are relatively more senescent display stronger invasiveness. Additionally, our risk model accurately predicts overall survival and reveals the multifaceted impact of cellular senescence on tumor metabolism and immunity. *In vitro* experiments demonstrate that *NME2*(Nucleoside Diphosphate Kinase 2) is a crucial SRG in ccRCC.

## 2 Materials and methods

### 2.1 Data acquisition

scRNA-seq data (GSE210038) downloaded from the Gene Expression Omnibus (GEO) database (https://www.ncbi.nlm.nih.gov/geo/), encompassed 7 ccRCC samples and 2 normal samples (Davidson et al., 2023). The bulk scRNA-seq data and clinical data were acquired from The Cancer Genome Atlas Program (TCGA-KIRC) (https://portal.gdc.cancer.gov/). To increase reliability, another KIRC dataset was obtained from the International Cancer Genome Consortium (ICGC) database (https://dcc.icgc.org/). Additionally, 8 samples (cancerous and adjacent tissues) were procured from individuals with ccRCC who received surgery at the First Affiliated Hospital of Harbin Medical University. All patients had advanced ccRCC, and their diagnoses were confirmed through hematoxylin and eosin (H&E) staining post-surgery. These samples were utilized for both Western blotting assays and immunohistochemical analysis to disclose the molecular mechanisms of ccRCC. To ensure the accuracy of the model, duplicate samples and those with survival time < 30 days were removed from scRNA-seq datasets.

### 2.2 The processing of scRNA-seq data

The Seurat package was employed to process the scRNA-seq expression matrix, and cells with mitochondrial gene expression exceeding 15% were removed. The AddModuleScore function was employed to estimate the senescence score based on the ‘FRIDMAN_SENESCENCE_UP’ gene set obtained from the Molecular Signatures Database (MSigDB). To further explore the interplay between cancer cells and TME, the CellChat package was leveraged to demonstrate the signaling pathways between cells. The CCPlotR package was responsible for visualization.

### 2.3 Construction of the SRSM

SRGs (FRIDMAN_SENESCENCE_UP) were obtained from the molecular signature database. To ensure the accuracy and reliability of the model, we established a machine-learning algorithm framework based on 101 combinations, which included partial least squares regression for Cox (plsRcox), Ridge, elastic network (Enet), CoxBoost, Lasso, StepCox, random survival forest (RSF), supervised principal components (SuperPC), generalized boosted regression modeling (GBM), and survival support vector machine (survivalSVM). 10-fold cross-validation was adopted to build SRSM. Besides, the TCGA-KIRC cohort was allocated into the training and testing cohorts in a 7:3 ratio, and the ICGC cohort served as an external validation cohort. To further investigate the prognostic value of SRSM, these cohorts were allocated into high-SRSM and low-SRSM groups based on the median SRSM.

### 2.4 Assessment of the prognostic value of SRSM

Kaplan-Meier curves and receiver operating characteristic (ROC) curves were leveraged to appraise the prognostic significance and accuracy of SRSM. Besides, univariate and multivariate Cox analyses were leveraged to assess the value of SRSM. Furthermore, the nomogram was developed to calculate the risk of ccRCC individuals.

### 2.5 Differential analysis and function enrichment analysis

To investigate the biological features of SRSM groups, the Limma package was adopted for differential analysis with log2FC > 1 and p < 0.05 as thresholds. Gene set enrichment analysis (GSEA) analysis was conducted using the GSEA and clusterProfiler packages. Results with p-values > 0.05 were discarded. Additionally, gene set variation analysis (GSVA) was employed to calculate the metabolism score with the GSVA package.

### 2.6 Evaluation of immune infiltration and drug sensitivity analysis

Four algorithms (CIBERSORT, EPIC, Quantiseq, XCell) were leveraged to assess the immune infiltration. Then, the abundance of TME components was calculated based on the ESTIMATE package. The oncoPredict package was adopted to determine the sensitivity to ccRCC chemotherapy drugs.

### 2.7 Genomic landscape alterations

To investigate the genomic variation across the high-SRSM and low-SRSM groups, copy number variations (CNV) were assessed based on the TCGA-KIRC and pan-cancer data. The maftools package was utilized to demonstrate the mutation of the top 15 genes. Besides, amplified and deleted regions were determined through the GISTIC 2.0 analysis (https://gatk.broadinstitute.org).

### 2.8 Cell culture and transfection

Human ccRCC cells (786O and A498) utilized were obtained from the Cell Bank of the Chinese Academy of Sciences (Shanghai, China). Both kinds of cells were cultured in high-glucose DMEM with 10% fetal bovine serum (Gibco, USA) and 1% streptomycin/penicillin (Thermo Fisher Scientific, USA) at 37°C and 5% CO_2_. The medium was refreshed daily, and cells were transfected upon reaching 70-80% confluence. Details are listed in **Supplementary Table 1**.

### 2.9 Wound-healing assay

We cultured cells in 6-well plates, and a sterile 200 µL pipette was utilized to scratch the layer when cells reached 90% confluence. Subsequently, cells were cultured in a serum-free medium. Images were captured at 0 and 24 hours.

### 2.10 Transwell assay

To observe the migration and invasion capability of cells, 786O and A498 were seeded at 2×10^4^ cells/well in the upper chamber with 200 µL of serum-free medium, while 800 µL of medium with 10% serum was filled in the lower chamber. Additionally, 50 mg/L Matrigel was PAVED in the upper chamber to perform an invasion assay. After the transwell plate was incubated for 48 hours, cells were fixed with 4% methanol solution and stained with 0.3% crystal violet.

### 2.11 Detection of cell viability and proliferation

As the CCK-8 assay kit (Biosharp, China) stated, absorbance at 450nm was read on a microplate reader (Thermo Fisher Scientific). EdU assay (Beyotime, China) was employed to examine cell proliferation. DNA synthesis was observed under a fluorescence microscope (Olympus, USA).

### 2.12 Real-time PCR analysis

TRizol reagent (Thermo Fisher Scientific) was utilized for RNA extraction from cells and tissues. The concentration and purity of RNA solution were checked using a NanoDrop 2000 spectrophotometer (NanoDrop Technologies, Wilmington, DC, USA). Next, 1 μg RNA was reverse transcribed into cDNA. SYBR Green mixture (YEASEN, Shanghai, China) was used for qPCR analysis on the StepOnePlus TM real-time PCR system (Thermo Fisher Scientific). GAPDH was utilized for sample normalization. The specific primer sequences are shown below.

*GAPDH*: Forward, 5′-GAGTCAACGGATTTGGTCGT-3′
Reverse, 5′-GACAAGCTTCCCGTTCTCAG-3′
*NME2*: Forward, 5′-GGACTTCTGCATTCAGGTTGGC-3′
Reverse, 5′-TGTAGTCAACCAGTTCTTC GGC-3′

### 2.13 Western blotting

Cells and tissues were lysed in RIPA lysis buffer (Beyotime Biotechnology Institute, Haimen, China) consisting of a mixture of PMSF and protease inhibitors. The protein concentration was then quantitated utilizing the BCA kit (Beyotime). 40 μg protein was separated through SDS-PAGE and then moved onto PVDF membrane. The PVDF membrane was blocked at ambient temperature for 1 hour using 5% skimmed milk and then incubated with the primary antibody *NME2* (Proteintech, 20493-1-AP) at 4°C overnight. Then, the membrane was washed and probed with the secondary antibody β-actin (Proteintech, 20536-1-AP) in a blocking buffer for 2 hours at ambient temperature. Finally, protein visualization was conducted with ChemiDoc-XRs + (Bio-Rad, Hercules, CA, USA).

### 2.14 Immunohistochemistry

4 μm sections were obtained from paraffin-embedded ccRCC and adjacent tissues. After deparaffinization and rehydration, the sections were incubated with H_2_O_2_ (3%) for 15 minutes at ambient temperature for antigen retrieval. Thereafter, these sections were probed with the primary antibody overnight at 4 °C and then with an HRP-conjugated secondary antibody for 1 hour. Finally, cell nuclei were stained with hematoxylin.

### 2.15 Statistical analysis

Based on R software and GraphPad Prism 8, data processing and analyses were made. Wilcoxon rank-sum test, Student’s t-test, Fisher exact test, and Kruskal-Wallis test were employed for comparisons among groups. Log-Rank test was adopted to analyze survival significance. All data were delineated as the mean ± SD. A p-value < 0.05 from two-sided tests implied statistical significance.

## 3 Results

### 3.1 scRNA-seq profiles of senescence in ccRCC

**Figure 1:**
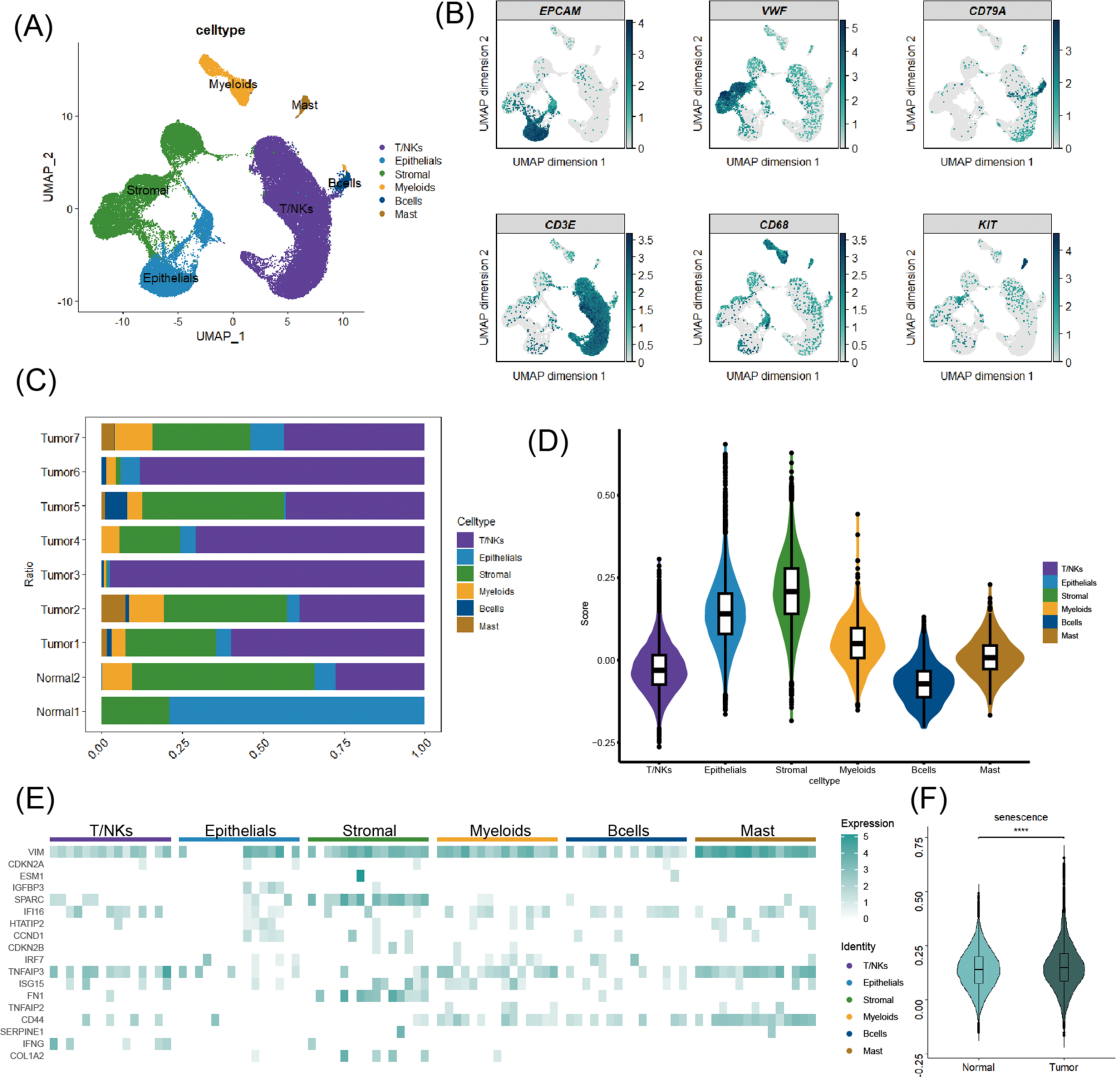
Overview of cellular senescence in scRNA transcriptome data of ccRCC. **(A)** Main cell types were illustrated by UMAP. **(B)** A heatmap of classical markers in ccRCC. **(C)** A bar chart of the proportion of different cell types. **(D)** Violin plots of the senescence of various cell types. **(E)** The heatmap of the distribution of SRGs in main cell types. **(F)** The distribution of cellular senescence between normal and tumor tissues ****p < 0.0001.

Based on classical cell markers (*EPCAM, VWF, CD79A, CD3E, CD68, KIT)*, single-cell transcriptome data were annotated into six major subpopulations (**Figure 1A**). Uniform Manifold Approximation and Projection (UMAP) illustrated the expression of these marker genes in scRNA-seq profiles (**Figure 1B**). Tumor samples had a higher infiltration of immune cells (**Figure 1C**). Stromal cells exhibited the highest levels of senescence (**Figure 1D**). Next, to investigate key senescence-associated features across the TME, we examined the expression of two representative markers: VIM, a classical marker of epithelial-mesenchymal transition (EMT) often associated with senescent cells, and *SPARC*, a core matricellular component of the senescence-associated secretory phenotype (SASP) crucial for extracellular matrix remodeling. We found that VIM was highly expressed across different cell types, suggesting a pervasive mesenchymal state within the ccRCC TME. Additionally, *SPARC* was highly expressed specifically in stromal cells (**Figure 1E**), aligning with their role as active SASP producers and highlighting their potential in shaping a pro-tumorigenic niche (**Figure 1E**). The senescence levels of ccRCC tissues were greatly higher than normal tissues among the TCGA cohort (**Figure 1F**). Epithelial cells were extracted from single-cell data, and their senescence scores were calculated. These epithelial cells were then divided into ‘high-senescence’ and ‘low-senescence’ subgroups based on the median value of the senescence score across all epithelial cells (**Supplementary figures 1A, B**). Subsequently, differential and enrichment analyses conducted between these two subgroups showed that the differential genes associated with high senescence scores were enriched in epithelial-mesenchymal transition (EMT) and hypoxia (**Supplementary figure 1C**). Cell communication analysis showed that epithelial cells with high senescence scores had more connections with TME cells (**Supplementary figures 1D, E**). Signaling pathway analysis revealed that epithelial cells with high senescence scores exhibited stronger EGF signaling pathways than those with low senescence scores (**Supplementary figures 1F, G**).

Collectively, these findings revealed a landscape of cellular senescence within the ccRCC tumor ecosystem. The enhanced communication between highly senescent epithelial cells and the surrounding TME cells, coupled with the activation of pro-tumorigenic pathways such as EGF signaling, suggests that the crosstalk stemming from senescent epithelial cells may actively contribute to ccRCC progression.

### 3.2 Construction of SRSM signature

**Figure 2:**
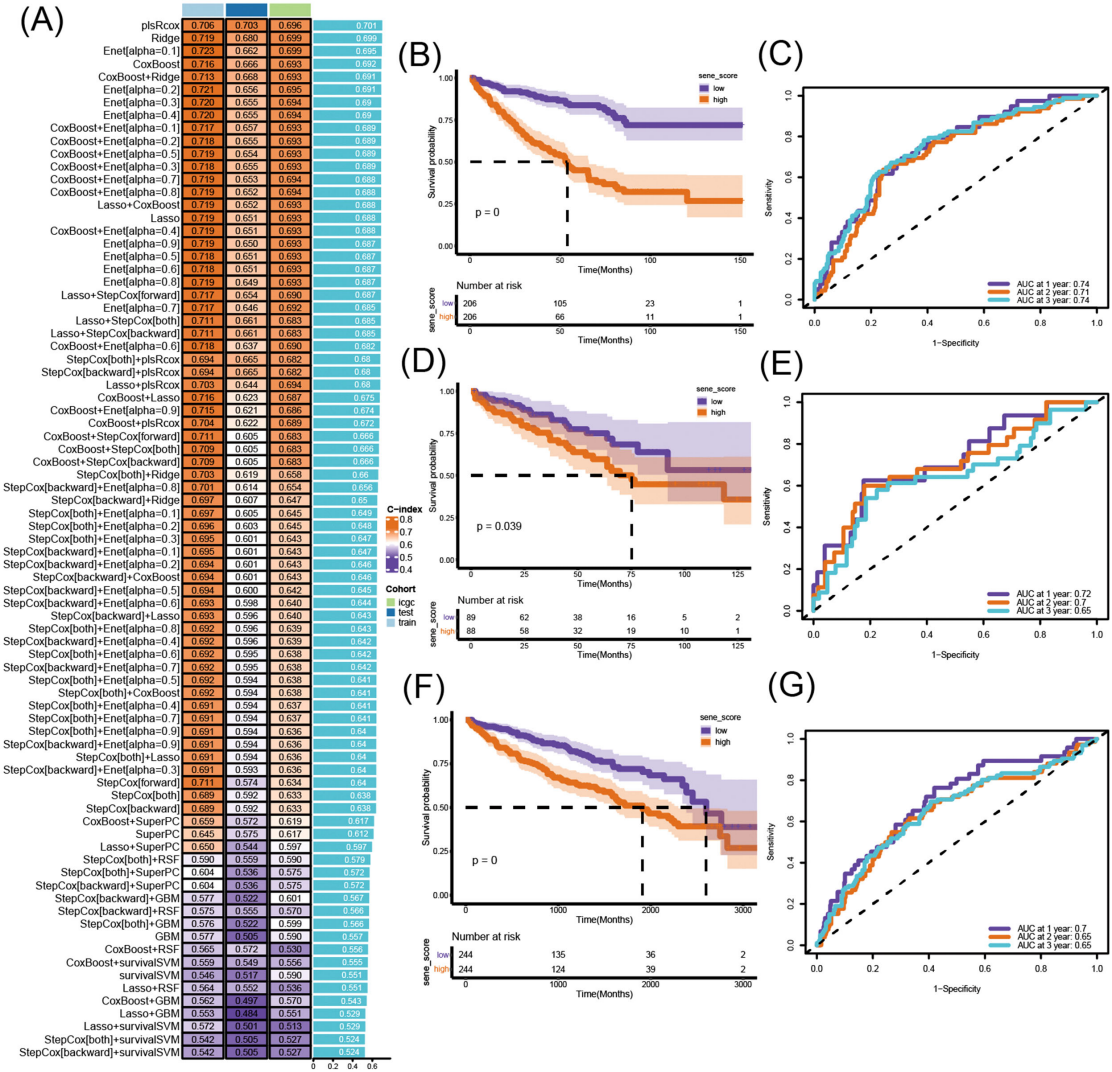
Construction of SRSM. **(A)** A heatmap displayed the optimal SRSM. **(B)** A K-M curve of the training cohort. **(C)** A ROC curve in the training cohort. **(D)** A K-M curve of the testing cohort. **(E)** A ROC curve in the testing cohort. **(F)** A K-M curve of the ICGC cohort. **(G)** A ROC curve in the ICGC cohort.

10 machine learning methods were integrated to construct a framework for a risk prediction model. When the expression matrix and survival data were input for the three cohorts into the framework, the Lasso+StepCox [both] algorithm, which achieved the highest average C-index while selecting the optimal number of genes, generated the optimal model (**Figure 2A**). In comparison, the model achieved the best prediction with the least genes. whereas the Lasso + StepCox [both] model incorporated only 9 genes(*ALDH1A3, CREG1, ESM1, MMP1, NME2, RHOB, SERPINB2, TNFAIP2, TSPYL5*), yet achieved comparable predictive efficacy. To validate SRSM, each cohort was divided into two groups based on the median value for survival analysis. The result yielded that the survival time of high-SRSM cohorts was markedly lower than that of the low-SRSM cohort (**Figures 2B, D, F**). In multiple external validation sets, the AUC values of the models ranged from 0.65 to 0.74, showing robust prognostic discriminant power (**Figures 2C, E, G**).

### 3.3 Evaluation of SRSM

**Figure 3:**
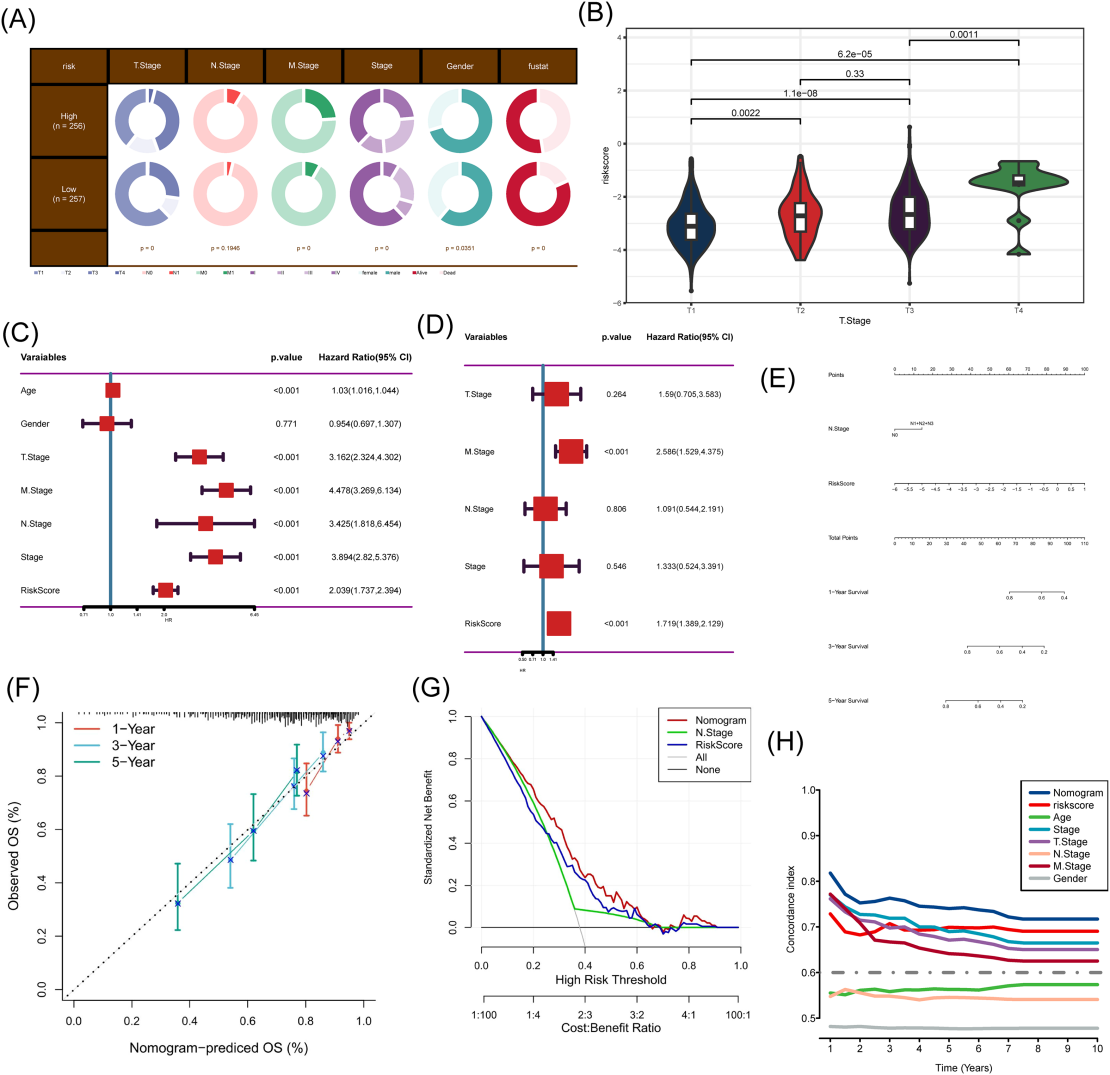
The validation of the SRSM. **(A)** Analysis integrating clinical information. **(B)** The distribution of SRSM across various T stages. **(C)** Univariate analysis in TCGA. **(D)** Multivariate analysis in TCGA. **(E)** The nomogram included SRSM and clinical characteristics. **(F)** A calibration curve of the nomogram. **(G)** DCA plot of SRSM nomogram. **(H)** Time-ROC curves depicted the efficacy of the SRSM nomogram.

Based on clinical information, SRSM was significantly linked with TNM stages, survival status, and tumor stage in the TCGA cohort (**Figure 3A**). SRSM also demonstrated excellent risk prediction performance across the T stages (**Figure 3B**). Univariate and multivariate Cox analyses yielded that SRSM was a significantly independent predictor for ccRCC (**Figures 3C, D**). Subsequently, an SRSM nomogram integrating multiple clinical factors was constructed (**Figure 3E**). The calibration plot yielded that the SRSM nomogram was highly accurate in predicting survival of ccRCC patients (**Figure 3F**). The SRSM nomogram manifested superior discriminatory capacity in identifying high-risk individuals (**Figure 3G**). ROC curve illustrated that the SRSM nomogram performed best among various predictors (**Figure 3H**).

### 3.4 The landscape of metabolism for SRSM

Metabolism-related signatures reported in previous literature and associated with cancer progression were collected for ssGSEA (single-sample GSEA) analysis. Significant metabolic differences were revealed. The high-SRSM group exhibited stronger oxidative phosphorylation and cholesterol biosynthesis (**Supplementary figure 2A**). Besides, the GSEA analysis (**Supplementary figure 2B**) noted that EMT was enriched in the high-SRSM group. The G2M checkpoint is often activated when there are DNA mutations or damage in cells. The high score of SRSM group experienced more genetic mutations. Moreover, the extracellular matrix (ECM) biological processes were more active in the high-SRSM group, implying that stromal cells in the tumor might be associated with worse outcomes (**Supplementary figure 2C**).

### 3.5 Immune infiltration of SRSM groups

**Figure 4:**
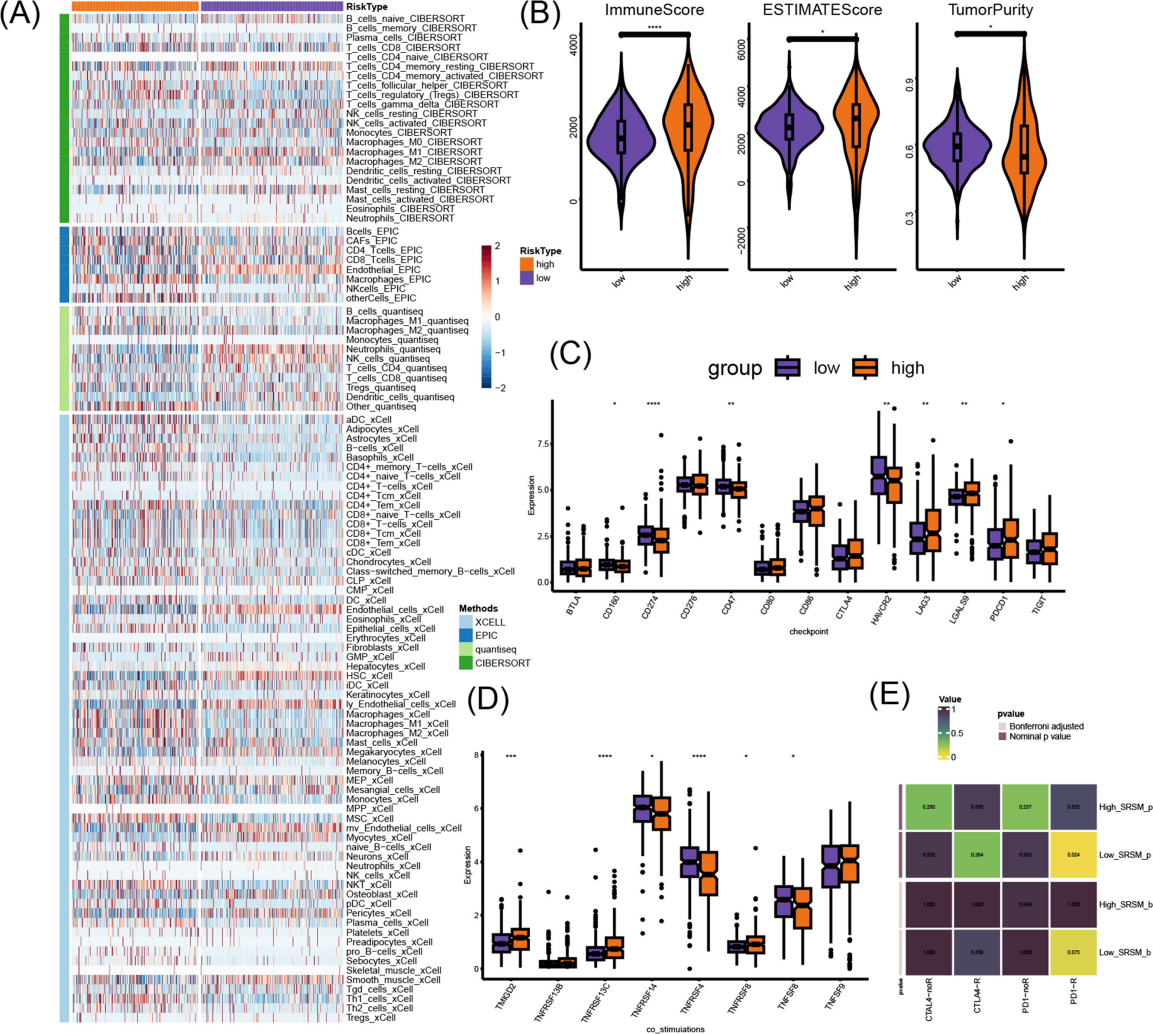
The immune infiltration impacted by SRSM. **(A)** The immune infiltration impacted by SRSM. **(B)** The distribution of TME abundance. **(C)** Different expression of immune checkpoint genes. **(D)** Different expression of co-stimulatory regulators. **(E)** Heatmap illustrated different responses to immune therapy. *p < 0.05, **p < 0.01, ***p < 0.001, ****p < 0.0001, ns, not significant.

The deconvolution analysis was conducted to understand the immune infiltration. As shown in the heatmap (**Figure 4A**), in the high-SRSM group, macrophage infiltration was relatively high, which might imply the presence of more senescent and dying cells that urgently needed to be cleared. Regulatory T cells (Tregs), which are classic immunosuppressive cells, also showed higher infiltration in the high-SRSM group, indicating an immunosuppressive TME in this group. Interestingly, fibroblasts, which belong to stromal cells, were more abundant in the high-SRSM group. This indicated that the crosstalk between fibroblasts and cancer cells within the TME was significant in tumor senescence. Additionally, the ESTIMATE package unveiled that the high-SRSM group possessed a more complex TME (**Figure 4B**). To investigate the impact of SRSM on ccRCC immunotherapy, immune checkpoints were compared in expression levels (**Figure 4C**). *CD274* expression was lower and *PDCD1* was higher in the high-SRSM group. The high-SRSM group demonstrated lower expression of costimulatory regulators (**Figure 4D**). Finally, low SRSM was linked with better responses to anti-PD-1 therapy (**Figure 4E**).

### 3.6 Genome landscape of different SRSM groups

CNV can, to some extent, represent the level of genetic mutations. The CNV map illustrated considerable differences between SRSM groups (**Supplementary figures 3A, B**). High-SRSM cohorts had more CNVs, mainly on chromosomes 3, 5, 6, 7, 9, and 14. Additionally, there were considerable differences in the mutations of the top 15 genes between SRSM groups (**Supplementary figures 3C, D**). Furthermore, high-SRSM cohorts were more sensitive to axitinib and sorafenib (**Supplementary figure 3E**).

### 3.7 Identification of the hub gene

To prioritize the most crucial gene within the SRSM signature for experimental validation, we employed a multi-step strategy. First, we assessed the expression and prognostic value of all 9 genes within the TCGA-KIRC cohort. Subsequently, to identify genes with broader oncogenic relevance, we performed a pan-cancer analysis of their copy number variation (CNV). This approach identified *CREG1*, *NME2*, and *TSPYL5* as frequently altered across multiple cancers. *MMP1, NME2, ESM1, TNFAIP2*, and *SERPINB2* were overexpressed in ccRCC tissues (**Supplementary figure 4A**). Additionally, they were overexpressed in various cancers. *CREG1, NME2*, and *TSPYL5* exhibited significant CNV across pan-cancer types (**Supplementary figure 4B**). Among these, *NME2* was selected for further functional validation due to its significant overexpression in ccRCC, its strong prognostic association, and most importantly, the compelling biological plausibility that its known role in nucleotide metabolism could directly underpin the enhanced oxidative phosphorylation phenotype observed in high-SRSM tumors.

### 3.8 *In vitro* experiments

**Figure 5:**
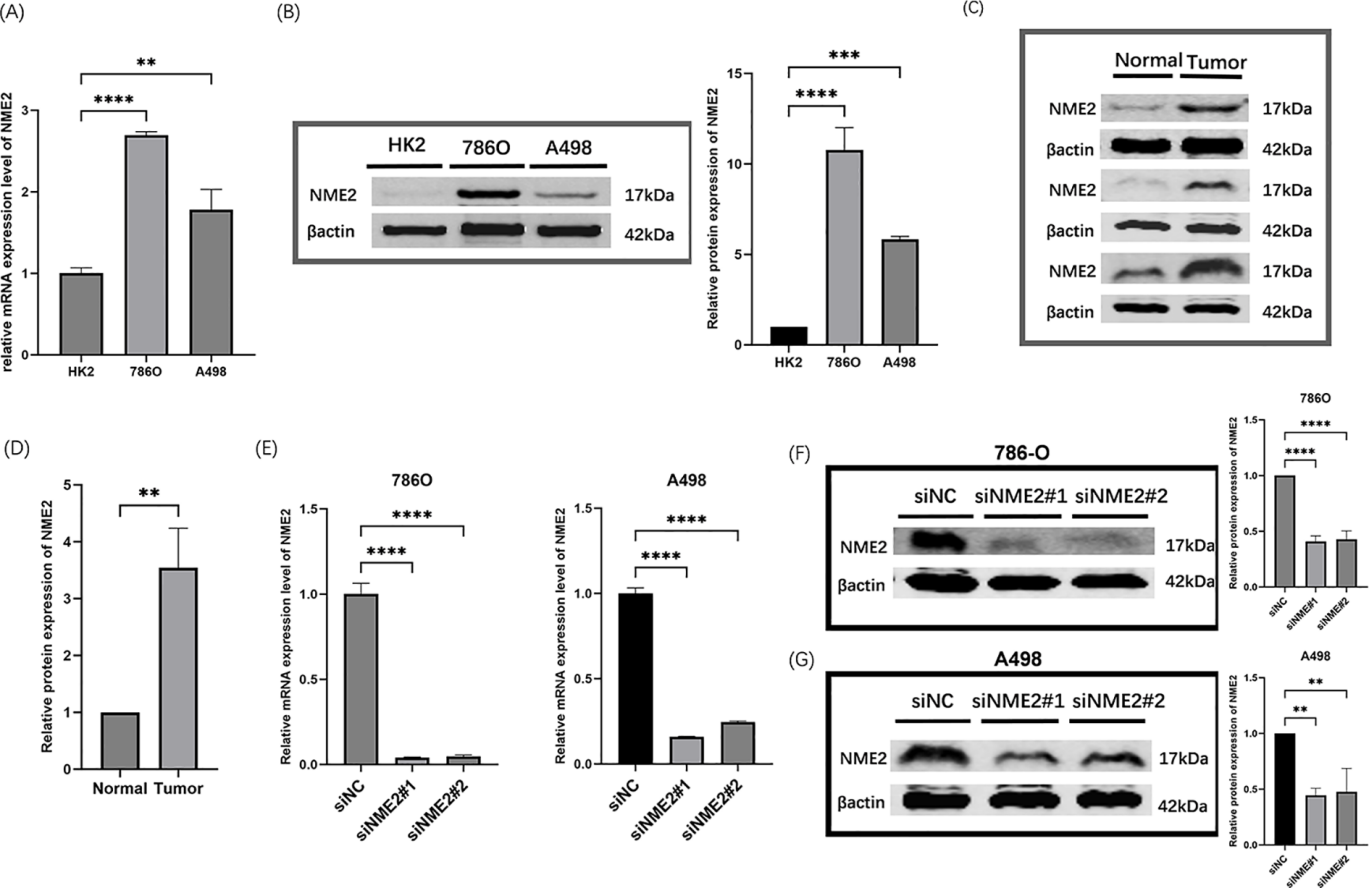
*In vitro* experiments. **(A, B)** Transwell assays evinced that NME2 knockdown curbed the invasion and migration of ccRCC cells. **(C-E)** CCK-8 and EdU assays demonstrated cell proliferation capacity. **(F, G)** Wound-healing assays elicited that NME2 facilitated the direct migration of ccRCC cells. *p < 0.05, **p < 0.01, ***p < 0.001, ****p < 0.0001, ns, not significant.

**Figure 6:**
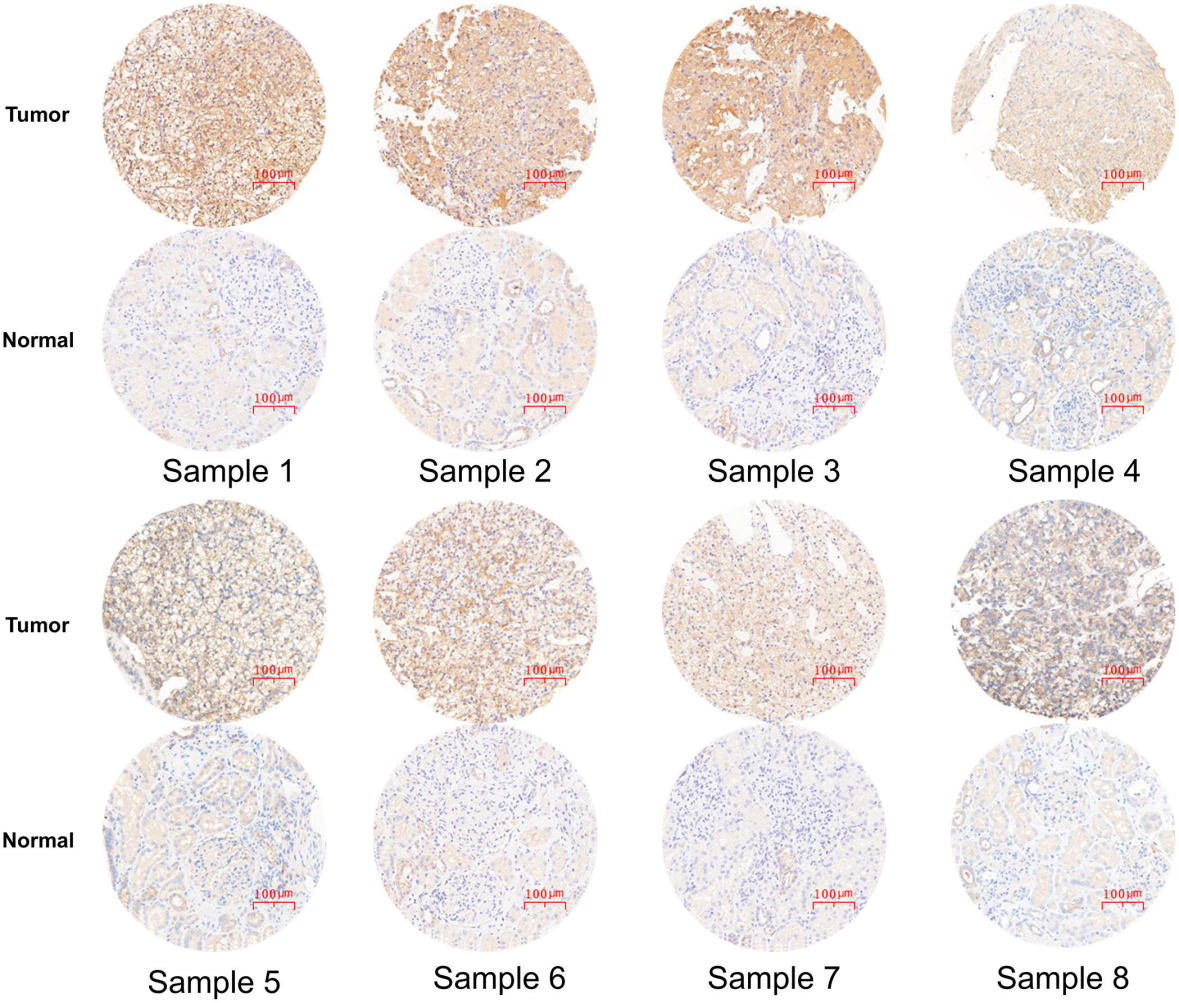
NME2 knockdown impedes ccRCC progression. CcRCC cell lines were transfected with siRNA to knock down NME2. qRT–PCR **(A)** and WB detection **(B)** of NME2 expression in ccRCC cell lines; qPCR and WB detection of NME2 knockdown efficiency in 786O and A498 cells **(C-E)**. Western blot confirmed the changes of NME2 expression in ccRCC tissues and adjacent non-tumor tissues**(F)**. *p < 0.05, **p < 0.01, ***p < 0.001, ns, not significant.

PCR and Western blot experiments revealed that *NME2* was significantly overexpressed in ccRCC cell lines (**Figures 5A, B**). Immunohistochemical staining of cancer tissues and adjacent tissues in 8 patients with ccRCC consistently elicited elevated *NME2* expression in tumor tissues (**Figure 6**), and Western blot analysis was performed on 3 pairs of ccRCC and adjacent normal tissues, a subset of the 8 pairs of samples mentioned in the “Data acquisition” section (Section 2.1). (**Figures 5C, D**).

**Figure 7:**
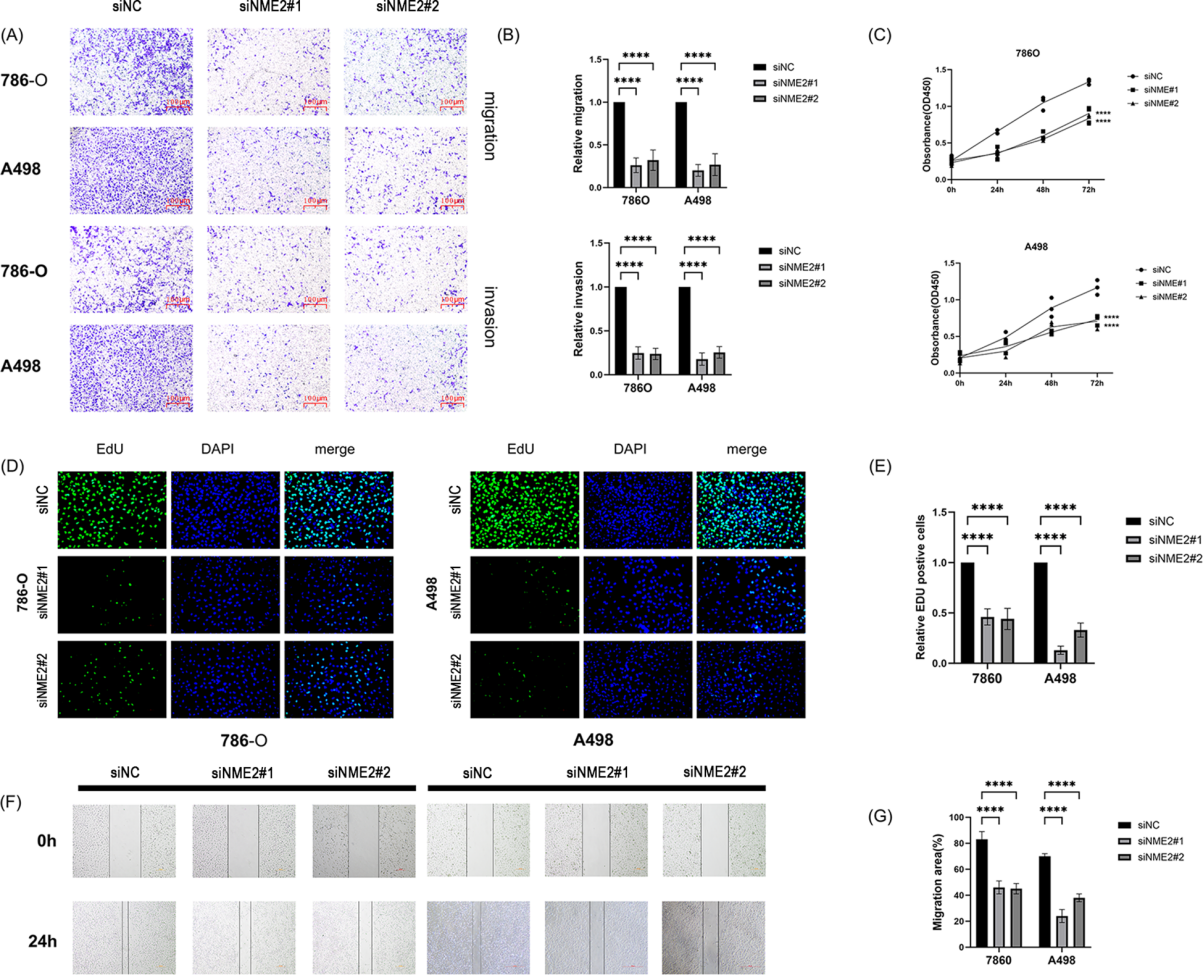
NME2 protein was strongly positive in ccRCC tissues and weakly in adjacent nontumor tissues ****p < 0.0001.

*NME2* expression was then knocked down in both 786O and A498 cell lines (**Figures 5E-G**). Transwell assays noted that *NME2* knockdown markedly reduced cell migration and invasion (**Figures 7A, B**). CCK-8 and EdU assays demonstrated that *NME2* knockdown significantly suppressed the viability and proliferation of ccRCC cells (**Figures 7C-E**). Subsequently,Wound healing assays showed notably reduced linear migration ability following *NME2* knockdown (**Figures 7F, G**).

## 4 Discussion

In this study, we first delineated the cellular senescence landscape within the ccRCC TME using scRNA-seq, which uncovered enhanced crosstalk between highly senescent epithelial cells and surrounding stromal/immune cells. Motivated by these findings and the limitations of existing prognostic models, we constructed a novel SRSM by employing a comprehensive machine-learning framework. This approach allowed us to efficiently distill the high-dimensional senescence-related information into a concise and powerful prognostic tool.

Currently, the primary modality for ccRCC is surgery accompanied by adjuvant chemotherapy (20). However, given the high heterogeneity of ccRCC, it tends to develop resistance to chemotherapy and immunotherapy (21). Therefore, identifying biomarkers and therapeutic targets is an urgent issue for the effective management of ccRCC patients (22).

Cellular senescence is an inevitable biological process for every cell (23). It can be triggered through various physical and chemical factors, such as oxidative stress, metabolism of toxic products, mitochondrial dysfunction, and activation of oncogenes (24). Cellular senescence helps to eliminate aging cells and toxic substances from the body and also induces the development of cancer and other diseases (25). Generally, oncogene activation induces cellular senescence, thereby limiting tumor growth (26). Mutations in von Hippel–Lindau (VHL) tumor suppressors are most common in ccRCC (27). In mouse models, VHL inactivation induces cellular senescence and tumorigenesis through p53 and HIF-mediated upregulation of pRB and p27 (28). Thus, the link between cellular senescence and ccRCC is complex. Additionally, tumor cells shape the TME through the regulation of SASP, making the TME of senescent cancer cells unique (29). SASP can recruit and activate cells in the TME (30). Senescent cells can motivate paracrine senescence in neighboring cells and secrete IL-8, which binds to the CXCR2 receptor on neighboring cells to induce and maintain their senescence. Senescent tumor cells also induce normal cell senescence through *VEGF, CCL2, TGFβ*, and *CCL20* (31). Tumor cell senescence may be intrinsic, but external chemotherapy can also induce tumor cell senescence (32). Numerous studies have revealed the potential link of cellular senescence with various cancers (25).

Unlike prior studies using single-algorithm approaches (e.g., Lasso-Cox), our multi-algorithm integration framework significantly enhanced prognostic accuracy (AUC ≥0.7 across cohorts) while minimizing gene signature size (9 genes vs. typical 15–30 genes). Consistent with integrative machine-learning models that simultaneously predict prostate-cancer diagnosis and biochemical recurrence, our multi-algorithm SRSM framework confirms that ensemble learning can compress high-dimensional molecular data into concise, clinically actionable signatures for precision oncology (33). This optimizes clinical applicability that significantly enhanced the precision and reliability of predictions. Through scRNA-seq data and datasets from GEO, TCGA-KIRC, and ICGC databases, we developed a robust and effective SRSM, which not only predicted clinical outcomes for patients and unveiled the profound impact of senescence on ccRCC prognosis and treatment response. Validation across multiple cohorts confirmed that higher SRSM scores were correlated with poorer survival rates, indicating that senescence markers can serve as indicators of aggressive tumor behaviors. Furthermore, SRSM demonstrated significant correlations with clinical characteristics, such as TNM staging and tumor grading, establishing its utility in clinical settings. The inclusion of SRSM in the comprehensive nomogram improved the accuracy of survival predictions, highlighting its relevance in personalized medicine. Metabolic analyses revealed that high-SRSM tumors exhibited increased oxidative phosphorylation and cholesterol biosynthesis, which supported rapid tumor cell proliferation and survival. These metabolic features may offer new therapeutic targets for patients with high-SRSM ccRCC. The complexity and heterogeneity of the TME in ccRCC present key challenges for therapeutic interventions, so the mechanisms driving tumor progression need to be elucidated. Our comprehensive evaluation of the immune TME revealed that high-SRSM tumors exhibited a complex TME, characterized by high infiltration of macrophages and Tregs and enrichment of fibroblasts. Elevated infiltration of macrophages and Tregs is typically associated with immunosuppression in the TME (34, 35). These characteristics suggest that an immunosuppressive environment may hinder effective immune responses. Interestingly, high-SRSM tumors showed lower expression levels of co-stimulatory molecules and higher levels of immune checkpoints, such as *PDCD1*, which may explain their poor responses to immunotherapy. Regarding genomic alterations, high-SRSM tumors had significantly higher levels of CNV, particularly on chromosomes 3, 5, 6, 7, 9, and 14, as well as a distinct mutational landscape. This genomic instability may drive tumor progression and resistance to standard therapies, which underscores the need for tailored treatment strategies. The concurrent upregulation of oxidative phosphorylation (OxPhos) and cholesterol biosynthesis in high-SRSM tumors, alongside an immunosuppressive TME characterized by Treg and macrophage infiltration, suggests a potential functional crosstalk between metabolic reprogramming and immune evasion. Enhanced OxPhos may provide the necessary bioenergetic support for the survival and function of immunosuppressive cells within the TME (36). Integrated multi-omics recently pinpointed the nucleotide-metabolism enzyme Cmpk1 as a monocyte-specific target in renal ischemia-reperfusion injury, underscoring that lineage-restricted metabolic hubs can be therapeutically leveraged to tame immune-driven renal disease—a rationale readily extendable to high-SRSM ccRCC (37). Likewise, ALDOC-driven aerobic glycolysis fuels neuroblastoma growth and chemo-resistance, illustrating that tumor-specific metabolic rewiring can couple proliferation with drug response—analogous to the OxPhos-centric aggressiveness of high-SRSM ccRCC (38). Furthermore, increased cholesterol biosynthesis not only sustains rapid membrane biogenesis for proliferating tumor cells but may also contribute to immune suppression through its precursors, oxysterols, which can modulate immune cell function and receptor signaling (39). The pro-tumorigenic role of *NME2*, identified in our study, might sit at the nexus of this interplay. Given its involvement in nucleotide metabolism, *NME2* overexpression could potentially fuel both anabolic processes and the associated metabolic pathways, thereby simultaneously supporting tumor proliferation and fostering an immunosuppressive niche. This integrated perspective underscores that cellular senescence in ccRCC orchestrates a coordinated program encompassing both metabolic and immunological rewiring, which collectively drives disease progression and therapy resistance.Aligned with recent AI-in-oncology reviews, large-language-model-powered knowledge mining will increasingly expedite the translation of multi-omics and machine-learning signatures like our 9-gene SRSM into prioritized targets and mechanistic insight (40).

Among the candidate genes derived from the pan-cancer analysis (*CREG1, NME2, and TSPYL5*), *NME2* was prioritized for functional validation based on the following rationale: its significant overexpression and prognostic value in ccRCC, its high frequency of copy number variations across cancers, and most importantly, the compelling biological plausibility that its known role in nucleotide metabolism could directly underpin the enhanced oxidative phosphorylation phenotype observed in high-SRSM tumors.

In this study, *NME2* was identified as an important target gene in ccRCC. *NME2* belongs to the NME family, which comprises 10 isoforms, of which NME1 and *NME2* are considered potential transcription factors due to their highly similar sequences (41). Overexpression of the *NME2* protein can notably stimulate the proliferation of osteosarcoma cell lines (42). However, in gastric cancer cells, *NME2* shows an inhibitory effect on cell proliferation and invasion (43). This contradictory phenomenon highlights the dual role of *NME2*, suggesting that its influence on tumorigenesis may vary across cancer types. These findings emphasize the distinct roles of *NME2* in different cancer contexts, indicating the complexity of research and therapeutic strategies. However, there is a dearth of research on *NME2* concerning ccRCC. According to our data analysis, *NME2*’s tumor-promotive role in ccRCC may involve metabolic reprogramming, as high-SRSM tumors showed enhanced oxidative phosphorylation. Given *NME2*’s known function in nucleotide metabolism (43), it potentially fuels ccRCC progression via purine synthesis – a hypothesis warranting future isotope tracing studies. with higher expression levels in tumors and a significant correlation with shorter patient survival. *In vitro* experiments validated the impact of *NME2* knockdown on the proliferation and invasion of ccRCC cells. Additionally, immunohistochemistry clarified differences in *NME2* expression between normal and tumor tissues, providing strong experimental evidence for NME2 as a therapeutic target in ccRCC.Although we did not perform direct senescence assays, the observed reduction in cell proliferation upon *NME2* knockdown (a hallmark of senescence) is consistent with the predicted pro-senescence role of this gene within our model.

Looking forward, integrating large-language-model-driven literature mining and protocol optimization—as recently reviewed in Large language models in clinical trials: applications, technical advances, and future directions—could accelerate the prospective validation of SRSM and facilitate the design of subsequent multicenter trials in ccRCC (44).

Nevertheless, this paper also has certain limitations. First, the sample size is relatively small, and the model requires further validation in larger cohorts. Second, the functional experiments are relatively basic, and no *in vivo* animal studies are conducted, nor are the functions of other key genes in the model deeply explored. Third, In the present study, cellular senescence was inferred at single-cell resolution using the AddModuleScore algorithm based on the FRIDMAN_SENESCENCE_UP gene set. This approach reflects the transcriptional abundance of senescence-associated genes rather than providing direct evidence of senescent status. Owing to the lack of freshly isolated tumour material, we were unable to perform the gold-standard assays that definitively identify senescent cells, including SA-β-galactosidase staining, senescence-associated heterochromatin foci (SAHF) visualisation, or quantitative measurements of canonical senescence markers such as p16/p21 protein quantification and other experiments. This absence of functional validation represents a major limitation of our work. In follow-up studies, we will prioritise the collection of viable single-cell suspensions or patient-derived organoids to integrate transcriptomic profiling with these established senescence read-outs, thereby consolidating the biological accuracy of the SRSM.Therefore, the specific effect of genes in the gene signature on tumor progression, and their roles in the TME still need to be further clarified.

## 5 Conclusion

Overall, the SRSM, constructed based on high-throughput data and machine learning techniques, has shown excellent performance in forecasting the survival of ccRCC patients. ccRCC patients with high score of SRSM exhibit stronger invasiveness, a more complex TME, and more genetic mutations. Subsequent *in vitro* experiments further confirm the impact of *NME2* on various malignant characteristics of ccRCC, highlighting its potential as an important target for ccRCC treatment and a promising candidate for future drug development.

## Data Availability

The original contributions presented in the study are included in the article/**Supplementary Material**. Further inquiries can be directed to the corresponding author.

## Glossary

RCC: Renal cell carcinoma
ccRCC: Clear cell renal cell carcinoma
SASP: senescence-associated secretory phenotype
TME: tumor microenvironment
SRGs: senescence-related genes
scRNA-seq: RNA-single-cell sequencing
GEO: Gene Expression Omnibus
TCGA: The Cancer Genome Atlas
ICGC: International Cancer Genome Consortium
plsRcox: partial least squares regression for Cox
Enet: elastic network
RSF: random survival forest
SuperPC: supervised principal components
GBM: generalized boosted regression modeling
survivalSVM: survival support vector machine
ROC: receiver operating characteristic
GSEA: Gene set enrichment analysis
GSVA: gene set variation analysis
CNV: copy number variations
UMAP: Uniform Manifold Approximation and Projection
EMT: epithelial-mesenchymal transition
ECM: extracellular matrix
Tregs: Regulatory T cells
VHL: von Hippel–Lindau.
