# Supplementary material for: Machine learning-derived cellular senescence index for predicting prognosis and drug sensitivity in patients with renal cell carcinoma
Source: Front Immunol. 2025 Dec 16;16:1727099. doi: 10.3389/fimmu.2025.1727099 (PMC12748256; doi:10.3389/fimmu.2025.1727099)
Supplement: Supplementary file 1 [file DataSheet1.pdf]

Supplementary table 1 siRNA sequence

	SS	AS
siNME2#1	5’-GCGAGAUCAUCAAGCGCUUTT-3’	5’-AAGCGCUUGAUGAUCUCGCTT-3’
siNME2#2	5’-GACCAAUCCAGCAGAUUCATT-3’	5’-UGAAUCUGCUGGAUUGGUCTT-3’

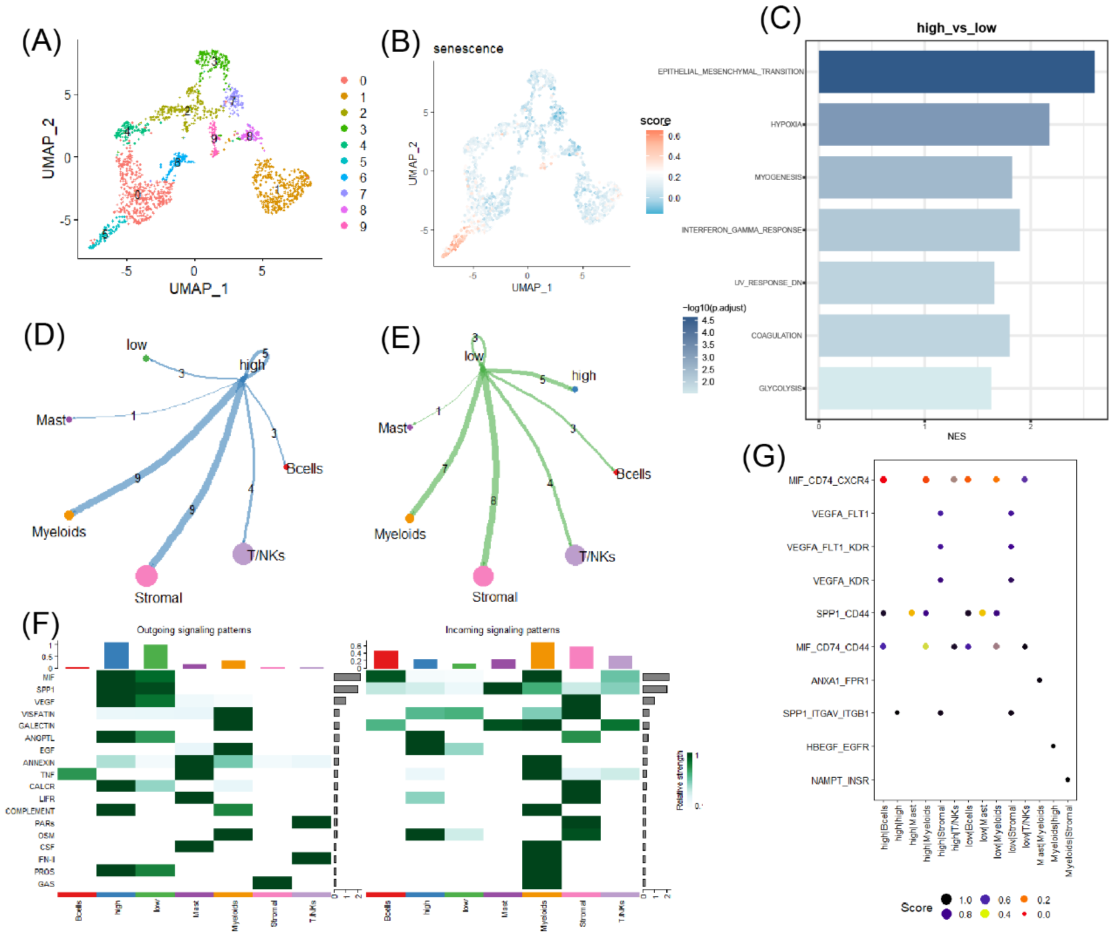

Supplementary figure 1 Single-cell RNA analysis of senescence. (A-B) UMAP plot showed cellular senescence in cancer cells. (B) Biological features of senescent cancer cells. (D-E) Cell-cell communication between cancer cells and TME. (F-G) The receptor-ligand analysis of senescent cancer cells.

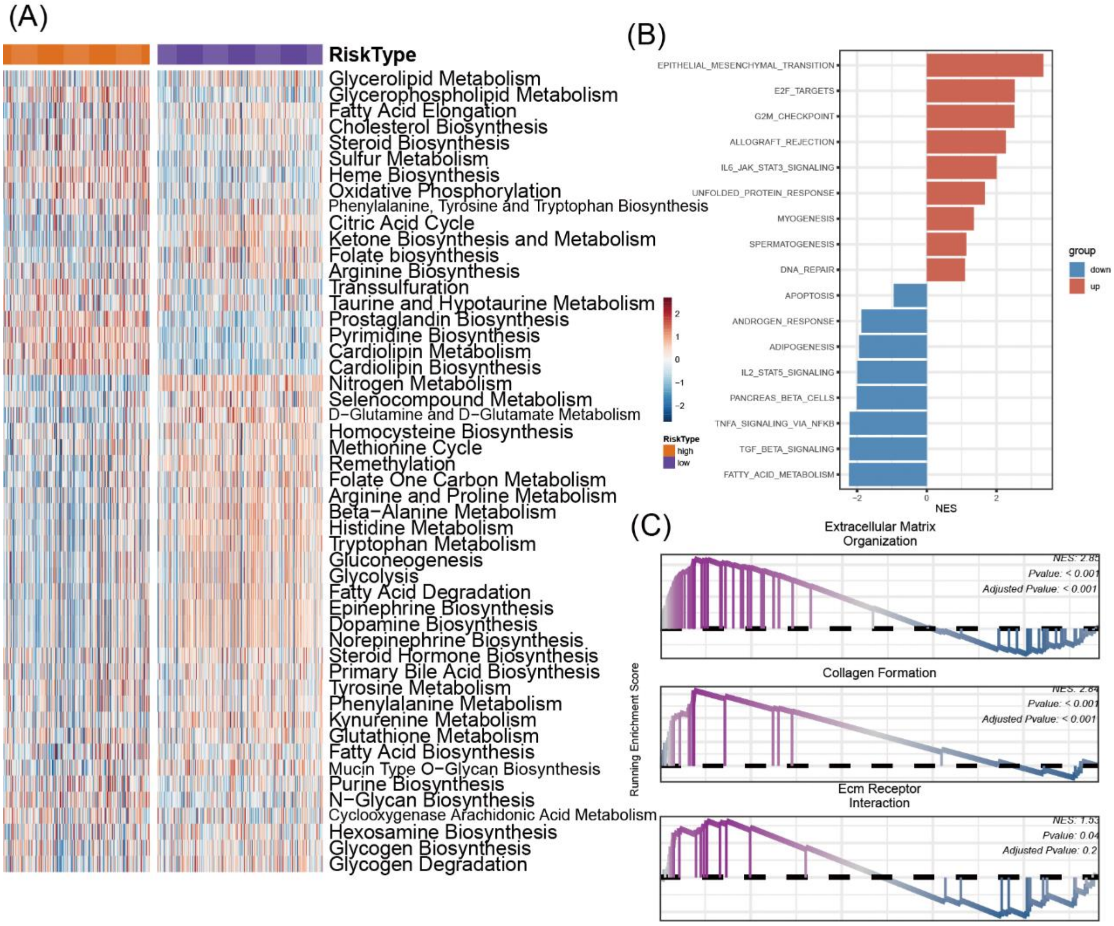

Supplementary figure 2 Biological features of different SRSM groups. (A) The landscape of metabolism impacted by SRSM. (B) A bar plot of biological characteristics across samples. (C) GSEA analysis of the differentially expressed genes.

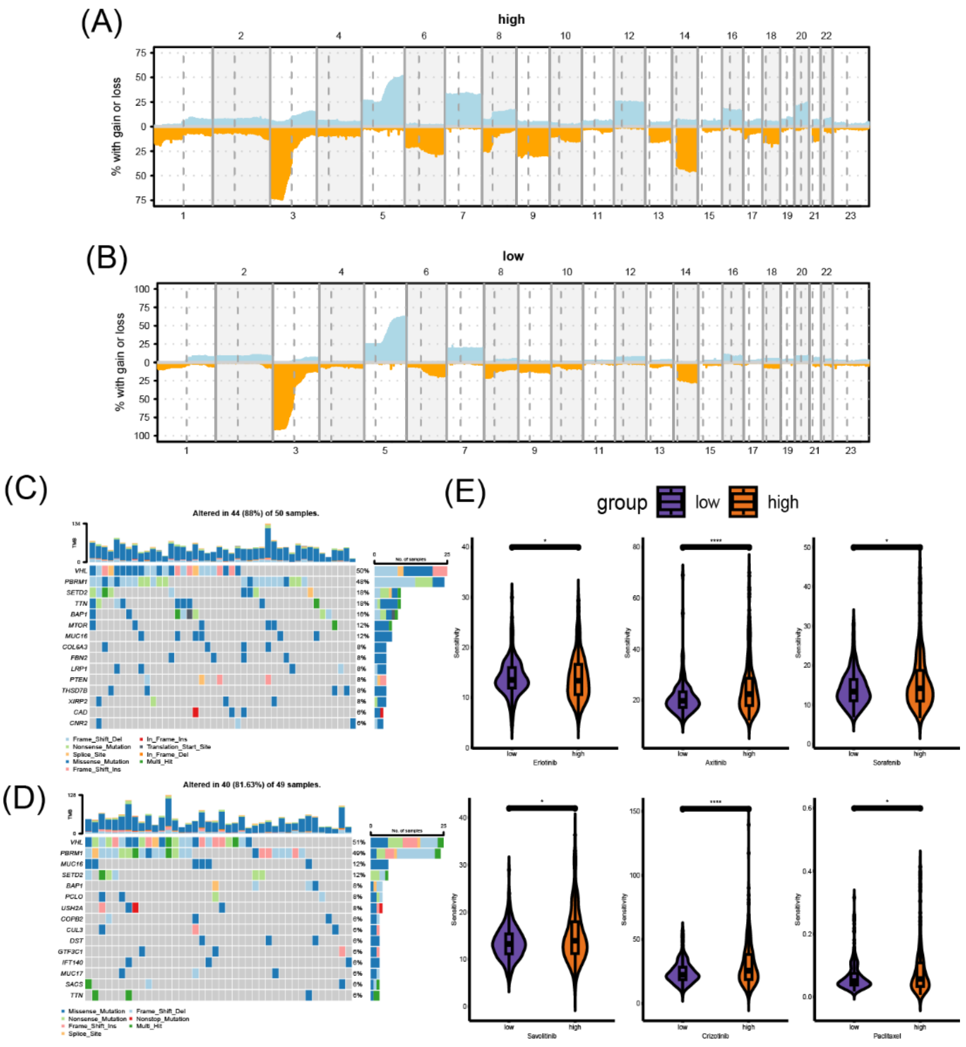

Supplementary figure 3 Genomic alterations and drug sensitivity affected by SRSM. (A-B) CNV difference between SRSM groups. (C-D) SNP difference between SRSM groups. (E) Response to different chemotherapeutic drugs in the SRSM groups. *p < 0.05, **p < 0.01, ***p < 0.001, ns, not significant.

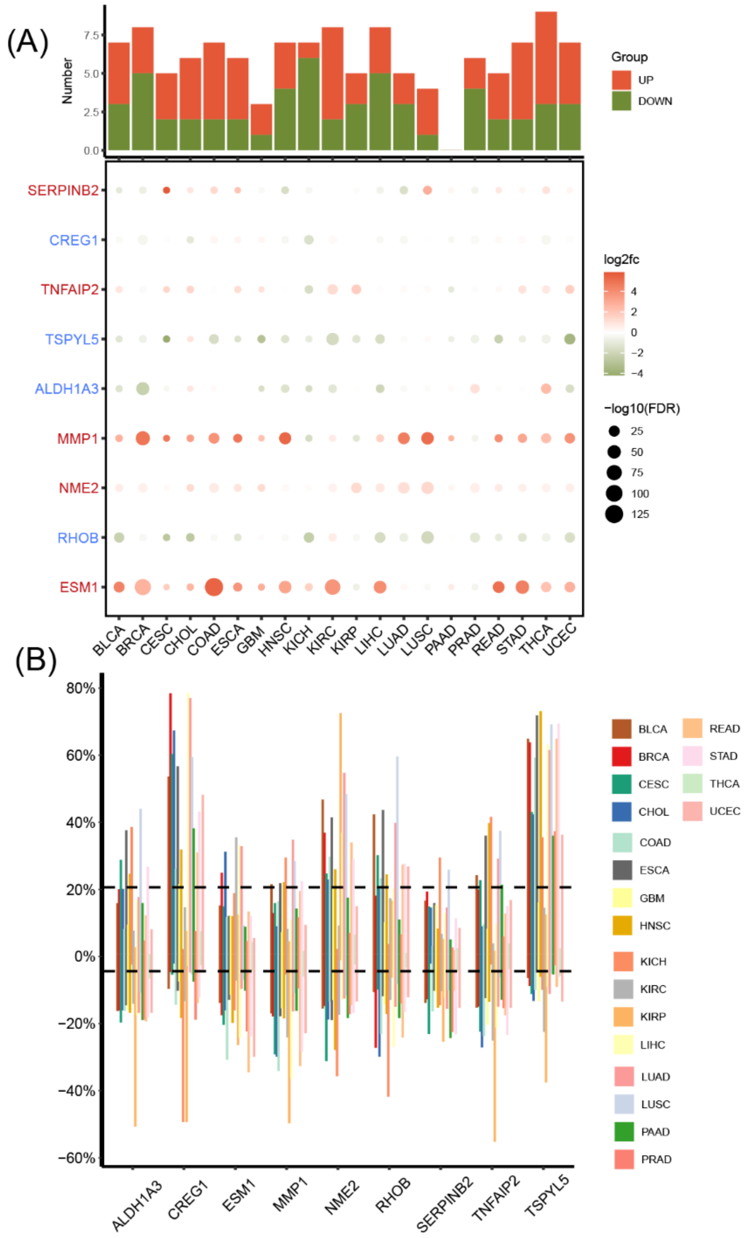

Supplementary figure 4 Identification of NME2. (A) The expression of genes in SRSM across pan-cancer. (B) The CNV of genes in the SRSM across pan-cancer.

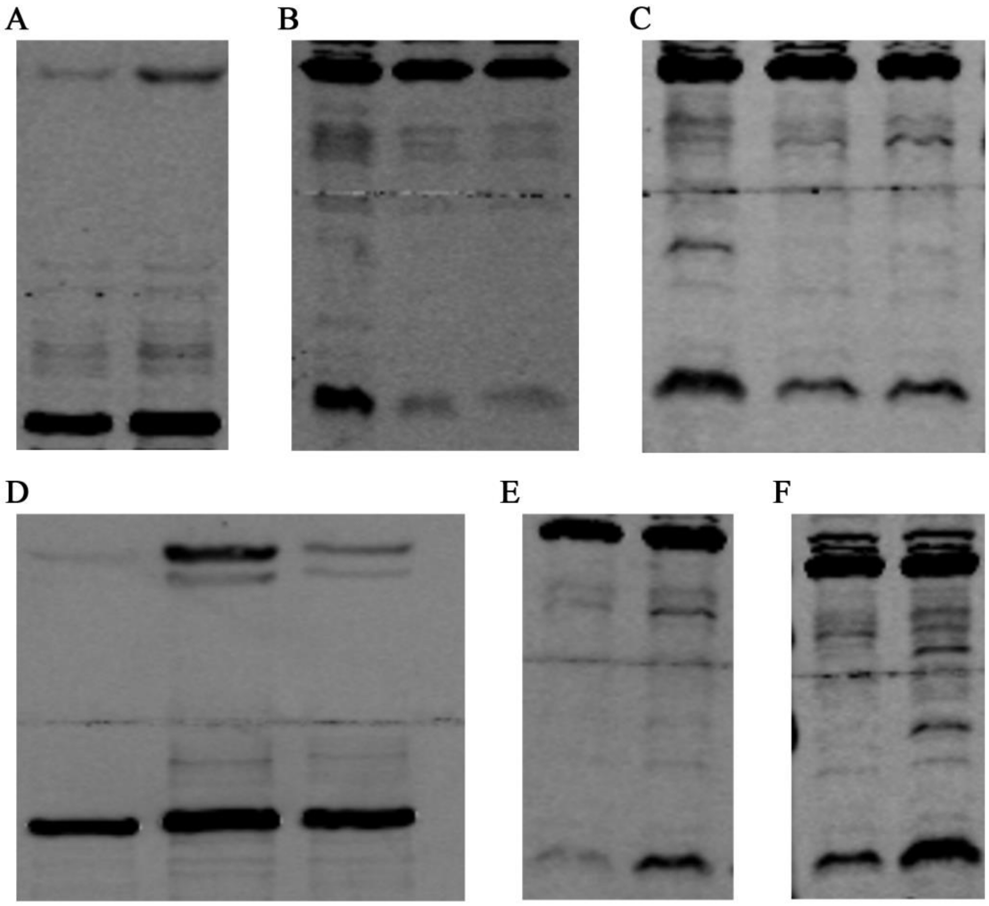

Supplementary figure 5 Western blot images of renal tissue cytokines. (A) WB results of NME2 in renal cell carcinoma tissues-1. (B) WB results of NME2 knockdown in 786O cell line. (C) WB results of NME2 knockdown in A498 cell line. (D) WB results of NME2 in HK2, A498 and 786O cell lines. (E) WB results of NME2 in renal cell carcinoma tissues-2. (F) WB results of NME2 in renal cell carcinoma tissues-3.
